# Evaluation of the Pathogenic-Mixed Biofilm Formation of *Pseudomonas aeruginosa*/*Staphylococcus aureus* and Treatment with Limonene on Three Different Materials by a Dynamic Model

**DOI:** 10.3390/ijerph19063741

**Published:** 2022-03-21

**Authors:** Edvige Gambino, Angela Maione, Marco Guida, Luisa Albarano, Federica Carraturo, Emilia Galdiero, Valeria Di Onofrio

**Affiliations:** 1 Department of Biology, University of Naples “Federico II”, 80126 Naples, Italy; edvige.gambino@unina.it (E.G.); angela.maione@unina.it (A.M.); marco.guida@unina.it (M.G.); luisa.albarano@szn.it (L.A.); federica.carraturo@unina.it (F.C.); 2 Department of Sciences and Technologies, University of Naples “Parthenope”, 80143 Naples, Italy; valeria.dionofrio@uniparthenope.it

**Keywords:** biofilms, *Pseudomonas aeruginosa*, *Staphylococcus aureus*, mixed infections, CDC biofilm reactor, continuous flow, limonene, gene expression

## Abstract

Background: Biofilms have been found growing on implantable medical devices. This can lead to persistent clinical infections. The highly antibiotic-resistant property of biofilms necessitates the search for both potent antimicrobial agents and novel antibiofilm strategies. Natural product-based anti-biofilm agents were found to be as efficient as chemically synthesized counterparts with fewer side effects. In the present study, the effects of limonene as an antibiofilm agent were evaluated on *Pseudomonas aeruginosa* and *Staphylococcus aureus* biofilm formed on different surfaces using the CDC model system in continuous flow. The *flgK* gene and the *pilA* gene expression in *P. aeruginosa,* and the *icaA* gene and *eno* gene in *S. aureus,* which could be considered as efficient resistance markers, were studied. Methods: Mono- and dual-species biofilms were grown on polycarbonate, polypropylene, and stainless-steel coupons in a CDC biofilm reactor (Biosurface Technologies, Bozeman, MT, USA). To evaluate the ability of limonene to inhibit and eradicate biofilm, a sub-MIC concentration (10 mL/L) was tested. The gene expression of *P. aeruginosa* and *S. aureus* was detected by SYBR Green quantitative Real-Time PCR assay (Meridiana Bioline, Brisbane, Australia). Results: The limonene added during the formation of biofilms at sub-MIC concentrations works very well in inhibiting biofilms on all three materials, reducing their growth by about 2 logs. Of the same order of magnitude is the ability of limonene to eradicate both mono- and polymicrobial mature biofilms on all three materials. Greater efficacy was observed in the polymicrobial biofilm on steel coupons. The expression of some genes related to the virulence of the two microorganisms was differently detected in mono- and polymicrobial biofilm. Conclusions: These data showed that the limonene treatment expressed different levels of biofilm-forming genes, especially when both types of strains alone and together grew on different surfaces. Our findings showed that limonene treatment is also very efficient when biofilm has been grown under shear stress causing significant and irreversible damage to the biofilm structure. The effectiveness of the sanitation procedures can be optimized by applying antimicrobial combinations with natural compounds (e.g., limonene).

## 1. Introduction

In natural environments, biofilms are the predominant mode of microbial growth and are frequently associated with persistent clinical infections. A biofilm is defined as a microbial community that adheres to biotic and abiotic surfaces. These communities are an aggregate of microorganisms organized in one or more layers that can attach to different types of surfaces such as food production equipment, creek stones, piping, external surfaces of marine vessels, wastewater treatment plants, air conditioning systems and cooling towers, prosthetic devices, and medical equipment such as endoscopes and colonoscopes and dental irrigation units [1]. In the medical field, biofilms play an important role in about 80% of microbial infections (bacterial vaginosis, fibrotic pneumonia, tract infections, infections of the tympanic cavity, chronic wounds, plaque formation, dental inflammation, endocarditis, and eye infections) [2].

As the biofilm is developed, this microbial mass has greater resistance to external stresses such as dehydration, predatory grazing, radiation, and antimicrobial compounds, compared to planktonic cells. Biofilms can grow on a variety of medical devices such as prostheses, cardioverter defibrillators, urinary and vascular catheters, and cardiac devices, showing distinctive and different characteristics when grown in different environments, so numerous approaches are developed to cultivate and study biofilms under conditions that mimic the environment of interest [3]. Furthermore, multi-species biofilm formed by bacteria/bacteria or fungi/bacteria are clinically common and confer the commensal microorganisms with protection against antimicrobial therapies [4].

Investigations of microorganisms’ behavior in biofilms composed by different species may have a high impact for understanding infectious diseases and to develop new therapeutic strategies. Indeed, *Pseudomonas aeruginosa* and *Staphylococcus aureus* strains often responsible for nosocomial infections in immunocompromised individuals and the most problematic pathogens in cystic fibrosis infections, tend to form polymicrobial biofilms [5,6].

The co-infection is usually associated with a persistence in serious infection, despite an aggressive antibiotic treatment, being the biofilm formation, an important contributor to this recalcitrance. The polymicrobial infections not only are more virulent than single-species infections but also lead to a chronic stage of infection [7,8]. Indeed, the emergence and re-emergence of infectious diseases are mainly caused by the ability of bacteria to resist antibiotics. Furthermore, due to the toxic side effects of synthetic antibacterial products and the problems associated with biodegradation, currently research has been oriented to find effective alternatives. In this context, there is an urgent need for safe and effective antibacterial and antifungal alternative agents with few side effects also as possible anti-biofilm agents [9].

Natural compounds of plant origin exhibit important biological properties and represent an alternative to conventional antimicrobial treatments, due to their broad-spectrum activity against microorganisms, mainly due to the alteration of the microbial membrane and cell wall, with consequent loss of cytoplasmic material [10]. Essential oils (EO)s are a complex mixture of various components such as aromatic/aliphatic molecules, flavonoids, catechins, and terpenoids, with important applications in pharmaceutical, sanitary, cosmetic, and agricultural and food industries with broad-spectrum activities against bacteria, fungi, and viruses [11]. The mechanisms of EOs action depend on functional groups that work by crossing the cell membrane and consequently disturb its integrity or they deregulate the communication system between bacteria, thus causing them to lose their ability to coordinate the interactions between themselves and their environment to survive or finally acting on regulation of quorum sensing genes leading to an inhibition in biofilm formation, the main virulence factor of microbes [12,13].

Limonene (1-methyl-4-(1-methylethenyl-cyclohexene) is a monocyclic monoterpene, commonly found in nature as a constituent of several *citrus* essential oils and is the precursor of several monocyclic monoterpenoids. Limonene is a colorless liquid and exists as two optical isomers, d-limonene, which is the main compound in the essential oils of the peels of *Citrus* spp., and L-limonene, which is mainly found in the essential oils of *Pinus* and *Mentha* species. It is considered safe, presenting low toxicity to humans, an excellent solvent for cholesterol, and effective in the treatment of chronic heartburn or gastro-esophageal reflux disorder with an anticancer, antioxidant, and antidiabetic activity [14,15].

In our previous study about biofilm grown in vitro, we employed a CDC Biofilm Reactor^®^ to grow biofilms on different coupon materials continuously exposed to shear stresses and renewable nutrients to mimic conditions in natural environments in order to evaluate the effect of antimicrobial treatments [16,17]. The CDC Biofilm Reactor^®^ is a standardized in vitro model that allows the biofilm formation on individual coupons under flow, avoiding the limitations of a static way of growth [18].

Therefore, in the present study, we hypothesized that the limonene effects would act as a better antibiofilm agent to combat *Pseudomonas aeruginosa* and *Staphylococcus aureus* biofilm on different surfaces using the CDC model system under continuous flow conditions.

In addition, we investigated the flagellar gene (*flgK*), and pilin gene (*pilA*) expression in *P. aeruginosa,* and the inter cellular adhesion gene (*icaA*), and laminin-binding protein gene (*eno*) in *S. aureus,* which could be considered as efficient resistance markers for bacterial pathogens against antimicrobial agents and to compare the prevalence of biofilm-related genes and their ability to form biofilm on different surfaces [19,20].

## 2. Materials and Methods

### 2.1. Bacterial Strains, Growth Conditions, Chemicals, and Test Materials

**Scheme 1:**
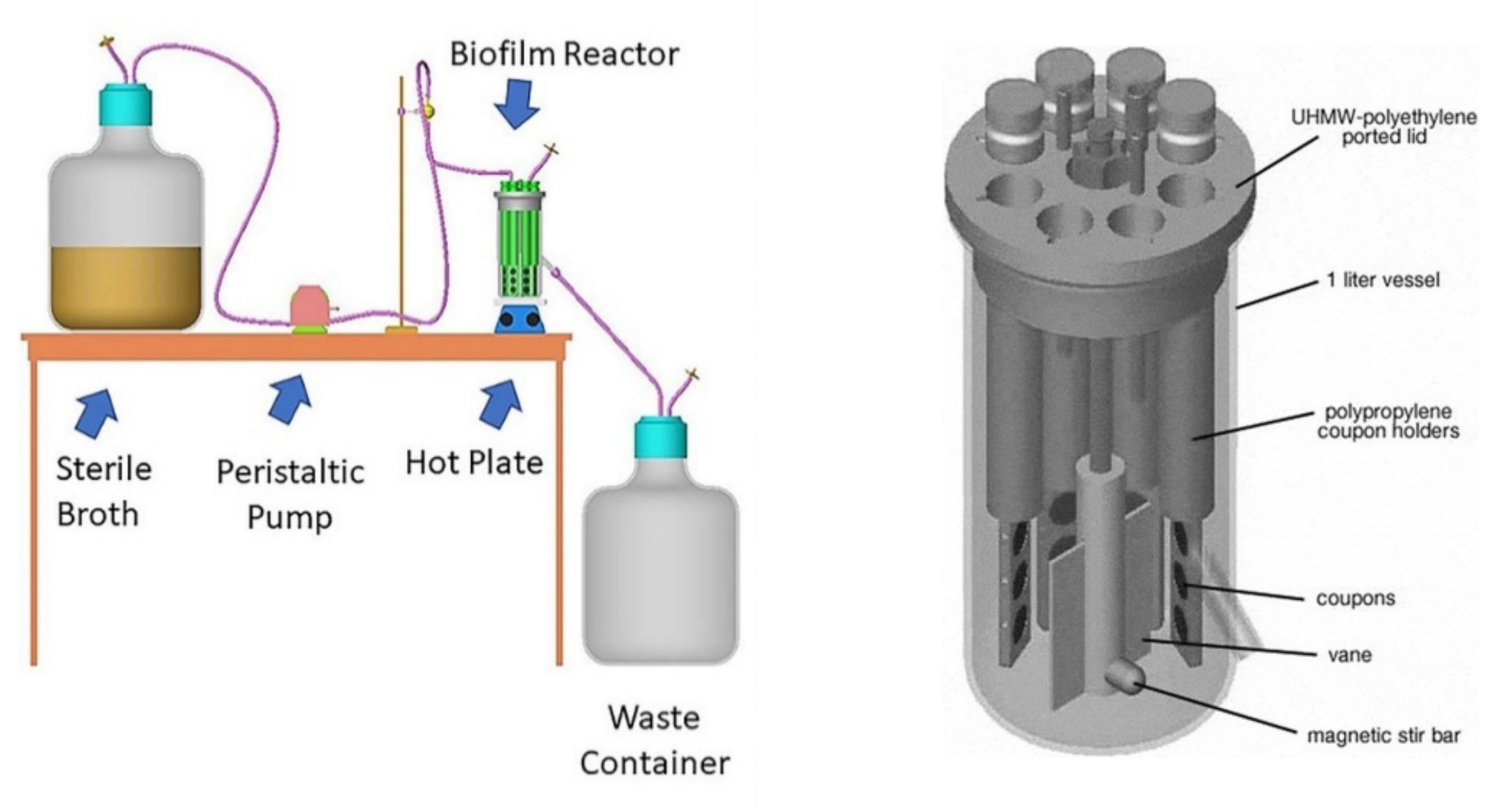
Schematic diagram of the CDC biofilm reactor (BioSurface Technologies Corp., Bozeman, MO, USA).

*P. aeruginosa* ATCC 10145 and *S. aureus* ATCC 6538 were stored in Bain Herat Infusion (BHI, Oxoid, Rodano, Italy) with 10% Glycerol at −80 °C. From an overnight pre-culture, bacteria were cultured in Triptone soya broth (TSB, Oxoid, Rodano, Italy) and incubated for 16–18 h at 37 °C, washed twice using sterile phosphate buffered saline (PBS, Oxoid, Rodano, Italy) and standardized to 10^6^ cells/mL or 10^8^ cells/mL for subsequent experiments. Limonene at 93.0% purity was purchased from Sigma Aldrich and a stock solution was prepared in PBS and tween 80 at 0.1% *v*/*v*. In this study, commercial coupons of polycarbonate, polypropylene, and stainless-steel (BioSurface Technologies Corp., Bozeman, MO, USA) were used (Scheme 1).

### 2.2. Susceptibility Test of Planktonic Cells

Minimal inhibitory concentration (MIC) and minimum bactericidal concentration (MBC) of limonene against *P. aeruginosa* and *S. aureus* were performed with a broth microdilution method according to Clinical and Laboratory Standards Institute [21]. Concentrations of limonene (S)-(-) (Sigma-Aldrich, Milan, Italy) (Formula Weight: 136.23 g/mol), ranging from 5 to 40 mL/L, were added to 96-well microplate containing the microorganism in TSB. The plate was incubated aerobically at 37 °C to 18–20 h. MIC values were determined as the lowest concentration inhibiting bacterial growth at 590 nm using a microplate reader (Synergy H4; BioTek Instruments, Inc., Santa Clara, CA, USA). MBC was determinate by inoculating 10 µL from the wells demonstrating no visible growth on TSA Tryptone soya agar (TSA, Oxoid) incubated for 24 h at 37 °C to count viable cells. The lowest concentration that led to ~99.9% decrease in CFUs/mL was considered the MBC.

### 2.3. Biofilm Formation and Inhibition/Eradication with Limonene in CDC Biofilm Reactor^®^ Model

Biofilms were grown on 3 coupons (polycarbonate, polypropylene, and stainless-steel) inserted into CDC biofilm reactor (Biosurface Technologies, Bozeman, MT, USA) [22]. In summary, an inoculum of 10^8^ CFU/mL of each microorganism for mono-species biofilms and a mixing of two (1:1 ratio) for dual-species biofilms, was added into the reactor containing 400 mL TSB. Biofilm was grown at 22 ± 1 °C with 125 ± 5 r/min stirring in batch conditions for 24 h, followed by a continuous flow of 11.7 ± 0.2 mL/min of a total of 20 L medium for another 24 h of incubation, in according to ASTM Standards E2562-12 (ASTM International. Standard Test Method for Quantification of *P. aeruginosa* Biofilm Grown with High Shear and Continuous Flow Using CDC Biofilm Reactor; ASTM International: West Conshohocken, PA, USA, 2007).

To evaluate the ability of limonene to inhibit and eradicate mono- and dual-species biofilm, a sub-MIC concentration (10 mL/L) was tested. Briefly, for the inhibition process, 4 mL of limonene was added to 400 mL of TSB in the reactor before the 24 h batch phase growth, while for the eradication process, the test substance was added, after a 48 h established biofilm, injecting into the reactor with a continuous flow phase to simulate the peak concentration of an antimicrobial during therapeutic regiment.

After a total of 48 h, the pump and baffle were turned off, coupons were removed and gently rinsed with 1 mL of PBS to remove loosely attached cells and scraped using a sterile scraper. The reduction in viable counts CFU (Colony Forming Units) was detected by plate assay. For this, after treatment, biofilm cell suspensions were serially diluted (ratio 1:10) in PBS and plated for mono-species biofilm onto TSA, while for dual-species biofilm, onto Baird Parker agar base (OXOID) to select *S. aureus* colonies, and Pseudomonas agar base (OXOID) to select *P. aeruginosa* colonies. The plates were incubated at 37 ± 2 °C for 24 h. The cell densities in log_10_ CFU/cm^2^ of surfaces of the coupons were calculated, following formulae were used as the ASTM Standard:

E2562-17 (5): Log_10_ (CFU/cm2) = Log_10_ (mean CFU/volume plated) × (volume scraped/surface coupon) × (dilution).

### 2.4. qRT-PCR Analysis

After a total of 48 h of development of mono- and dual-species biofilm on polypropylene, polycarbonate, and steel coupons, in the presence of limonene at sub-MIC concentration of 10 mL/L, bacterial mRNA was extracted and purified according to Direct-zol TM RNA Miniprep Plus Kit (ZYMO RESEARCH, Irvine, CA, USA). The same procedure was performed for bacterial mRNA extraction after the development of single and double species biofilms on polypropylene, polycarbonate, and steel coupons in the absence of limonene. This represented the control. cDNA was obtained by iScript™ cDNA Synthesis kit (Bio-Rad, Milan, Italy). The primers (Table 1) were synthesized by Biofab Research srl, Rome Italy. The gene expression of *P. aeruginosa* and *S. aureus* were detected by SYBR Green quantitative Real-Time PCR assay (Meridiana Bioline, Brisbane, Australia). The reaction was run on AriaMx Real-Time PCR instrument (Agilent Technologies, Inc., Santa Clara, CA, USA), according to the manufacturer’s instructions System thermal cycler, as follows: 10 min at 95 °C (1 cycle—cDNA denaturation); 15 s at 95 °C and 1 min at 60 °C (40 cycles—amplification); 15 s at 95 °C (1 cycle—final elongation); 1 cycle for melting curve analysis (60–95 °C) to verify the presence of one product. Agilent Aria 1.7 software (Agilent Technologies, Inc., Santa Clara, CA, USA) was used to measure fluorescence. The REST software was used (version n., software tool for relative expressions, Weihenstephan, Germany) to calculate the relative expression ratios from the quantification cycles (Cq) through a computation method corrected for efficiency (E) (Etarget ∆Cq target (mean control-mean sample)/reference ∆Cq reference (mean control-mean sample). For *S. aureus* and *P. aeruginosa* the expression of each gene was analyzed and normalized against *16S rRNA* and *rpoS*, respectively [23,24].

### 2.5. Statistical Analysis

The results were reported as the mean values and SDs obtained from three different observations. Two-way ANOVA followed by Tukey’s multi comparation test was performed for biofilm formation, inhibition, and eradication; *p*-values < 0.05 were considered significant. For molecular analyses, relative expression ratios greater than ± 1.5 were considered significant. Nonparametric Mann–Whitney test was applied to ∆Cq (Cq gene of interest—Cq reference) values between treated and control samples (biofilms grown in absence of limonene; n = 3). *p*-Values < 0.05 were considered significant. Statistical analyses were performed using GraphPad Prism Software (v9.00 for Windows, GraphPad Software, La Jolla, CA, USA, www.graphpad.com, accessed on 1 February 2021).

## 3. Results

To assess the rate of antimicrobial resistance to limonene we tested its activity against *S. aureus* and *P. aeruginosa.* As showed in Table 2, limonene had a MIC activity of 20 and 40 mL/L for the Gram-positive and Gram-negative bacteria respectively. It did not show an MBC activity toward neither of them confirming its bacteriostatic activity.

**Figure 1:**
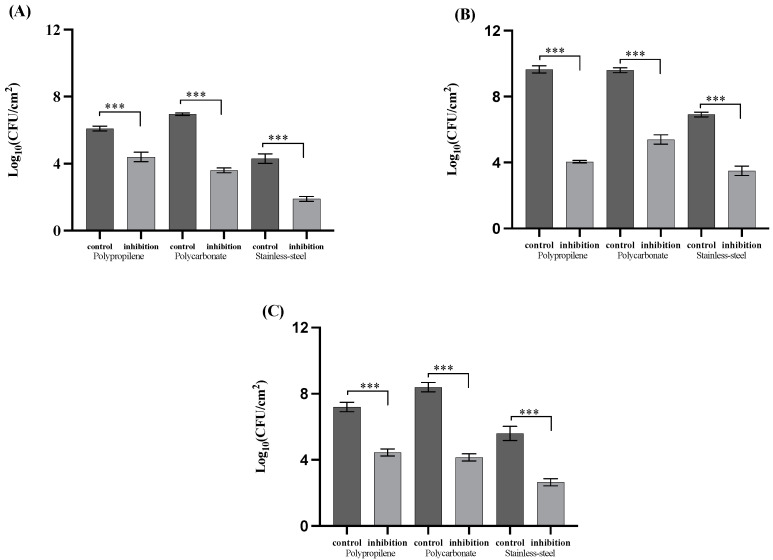
Comparison of limonene inhibition on mono- and polymicrobial mature biofilms ((**A**), *S. aureus* biofilm; (**B**), *P. aeruginosa* biofilm; (**C**), *S. aureus*/*P. aeruginosa* biofilm) grown in dynamic conditions in a CDC reactor on different materials (polypropylene, polycarbonate, and steel). Testing performed in triplicate. Error bars represent standard deviation. Statistical analysis was by two-way analysis of variance (ANOVA) with Tukey’s multi-comparation test (*** *p* value < 0.001).

**Figure 2:**
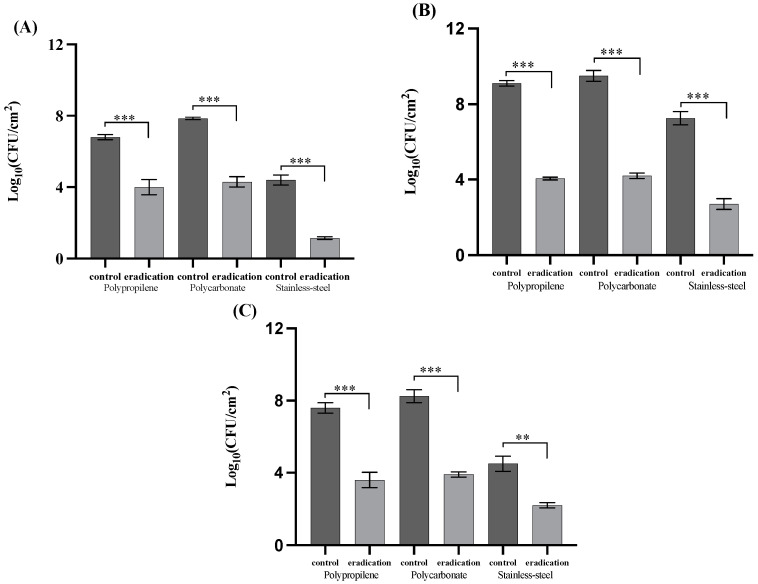
Comparison of limonene eradication on mono- and polymicrobial mature biofilms ((**A**), *S. aureus* biofilm; (**B**), *P. aeruginosa* biofilm; (**C**), *S. aureus*/*P. aeruginosa* biofilm) grown in dynamic conditions in a CDC reactor on different materials (polypropylene, polycarbonate, and steel). Testing performed in triplicate. Error bars represent standard deviation. Statistical analysis was by two-way analysis of variance (ANOVA) with Tuckey’s multi-comparation test (** *p* value < 0.01, *** *p* value < 0.001).

As shown in a previous paper [16], biofilms grown in a CDC reactor could be considered a valid surrogate for in vivo biofilms, and in Figure 1 and Figure 2, the capacity to form mono- and polymicrobial biofilms on all three coupons in a dynamic model for both microorganisms tested with differences between the three materials and the two microorganisms has been reported. *S. aureus* biofilm formation was of 10^6^, 10^7^, and 10^4^ CFU/cm^2^ on polypropylene, polycarbonate, and steel respectively. Limonene inhibited biofilm formation of about 10^4^ CFU/cm^2^ on polypropylene, polycarbonate, and 10^3^ CFU/cm^2^ on steel. Moreover, it eradicated mature biofilm of about 10^4^ CFU/cm^2^ on polypropylene, polycarbonate, and 10^2^ CFU/cm^2^ on steel. A better biofilm formation was observed for *P. aeruginosa* on polypropylene, polycarbonate coupons with 10^9^ CFU/cm^2^, and 10^6^ CFU/cm^2^ on steel coupon. When we treated these coupons with limonene, we observed a reduction in growth during inhibition of 10^4^ CFU/cm^2^ for polypropylene and steel, and 10^5^ CFU/cm^2^ for polycarbonate. The same values were observed when limonene was added on mature biofilm with a reduction in growth of about 10^4^ CFU/cm^2^ for all three different materials.

The mixed *S. aureus/P. aeruginosa* biofilm (Figure 1 and Figure 2) varies from 10^7^ CFU/cm^2^, 10^9^ CFU/cm^2^, and 10^6^ CFU/cm^2^ for polypropylene, polycarbonate, and steel, respectively. Moreover, limonene inhibited biofilm formation, decreasing growth up to 10^4^ CFU/cm^2^ for polypropylene, polycarbonate, and until 10^3^ CFU/cm^2^ for steel, with eradication at 10^4^ CFU/cm^2^ for both polypropylene and polycarbonate, and 10^3^ CFU/cm^2^ for steel coupons.

**Figure 3:**
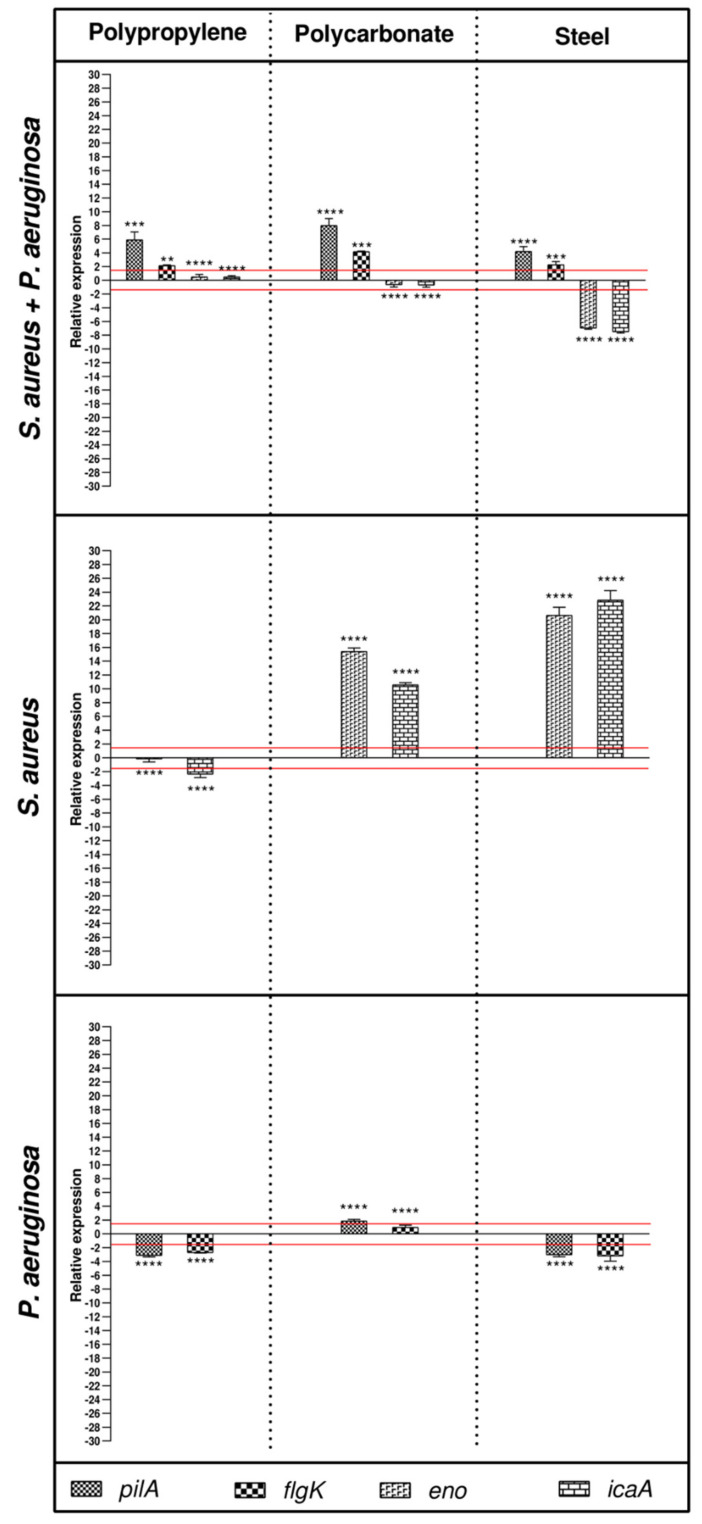
*Real-time qPCR* at 24 h of treatment. Histograms show the differences in the expression levels of 4 genes involved in biofilm formation and cell-cell communication. Bacterial biofilm was grown in the presence of limonene at sub-MIC concentration. Fold differences greater than ±1.5 (see red dotted horizontal guidelines at values of +1.5 and −1.5) were considered significant (see Table 3 for the values). Sisak’s test (**** *p* < 0.0001; *** *p* < 0.001; ** *p* < 0.01).

In order to detect the expression of genes related to biofilm formation and cell–cell communication during the inhibition treatment with limonene, we use a *Real-Time qPCR*. Specifically, the relative expressions of *eno* and *icaA*, and *pilA* and *flgK* genes were evaluated for *S. aureus* and *P. aeruginosa*, respectively. After 24 h of *S. aureus* and *P. aeruginosa* biofilms in combination treatment, both *pilA* and *flgK* were up-regulated, respectively, to control (untreated biofilms) considering all substrate. In addition, *pilA* gene was up-regulated by limonene in biofilms grown on polypropylene (5.91-fold), polycarbonate (7.99-fold), and steel (4.21-fold) (Table 3). The steel data were statistically significant compared to those observed for polypropylene (*p* < 0.01) and polycarbonate (*p* < 0.0001), which, in turn, were statistically significant between them (*p* < 0.001). *flgK* gene was up-regulated by limonene in biofilms grown on polypropylene (2.16-fold), polycarbonate (4.18-fold), and steel (2.27-fold) (Table 3). The polycarbonate data were statistically significant compared to those showed for polypropylene (*p* < 0.01) and steel (*p* < 0.001) (Figure 3 and Table 3). In contrast, *eno* (*p* < 0.0001) and *icaA* (*p* < 0.0001) genes were down regulated only using steel as substrate with a decrease of 6.98-fold and 7.50-fold, respectively (Figure 3). After 24 h of *P. aeruginosa* biofilm treatment, expression levels of *pilA* gene changed for all substrate, whereas the *flgK* gene was targeted only by treatment on polypropylene and steel. Especially, *spepilA* and *flgK* genes were down-regulated on polypropylene and steel (3.13-fold and 2.72-fold, respectively; Table 3). Furthermore, the *pilA* gene (*p* < 0.0001) was up-regulated by treatment on polycarbonate (1.88-fold). Finally, after 24 h of *S. aureus* biofilms treatment, expression levels of *icaA* gene changed for all substrate, whereas the *eno* gene was targeted only by treatment on polycarbonate and steel (Figure 3). In particular *eno* and *icaA* genes were up-regulated on polycarbonate and steel (15.41-fold and 20.65-fold, respectively; Table 3). Furthermore, *icaA* gene (*p* < 0.0001) was down-regulated by treatment on polypropylene (2.38-fold).

## 4. Discussion

*P. aeruginosa* and *S. aureus* are often present in polymicrobial infections simultaneously, and several studies have shown that their interactions are important for virulence, disease progression, and treatment outcome [27,28]. Since *P. aeruginosa* and *S. aureus* often adhere to colonize medical devices and aggregate to form mature biofilms [29,30], we believe it is important to study the interactions between these species.

In this study, *P. aeruginosa* and *S. aureus* planktonic and mono/polymicrobial biofilm growth was shown to be inhibited by limonene.

Biofilm, surface-associated microbial communities, has many negative effects, including medical device-related infections. Biofilm formation represents a protected mode of growth that causes bacterial cells, such as pathogenic microorganism, to become less susceptible to high concentrations of antibiotics and so are able to survive in hostile environments. This is one of the causes of treatment failure and infection recurrence.

Various factors affect the susceptibility of the pathogens in a biofilm as activation of biofilm phenotype, stress responses, and decreased penetration of antimicrobial agents due to the EPS matrix. Alternative strategies or development of new antimicrobial agents showing activity against pathogens in a biofilms way of growth are of great practical significance. To know the nature of biofilms by understanding the composition of the microbial communities, which form on implanted devices, is the first important step in assessing the impact of biofilm and estimating antimicrobial treatments.

Essential oils are widely used as a possible alternative therapy for their antimicrobial effects. Different essential oils of plants have been shown to have an antimicrobial activity and have been used as topical and oral antimicrobial treatments [31,32]. Moreover, the inhibitory and eradicating activity of essential oils has already been demonstrated to be effective against biofilms formed by bacteria of medical relevance [33].

Many studies have shown the antibacterial activity of limonene, one of the major ingredients of essential oils in various plants and seeds [34,35,36].

In this study, coupons assemblies with different material and roughness were inserted into a CDC Biofilm Reactor and were compared to value not only the mono- and polymicrobial biofilm formation of *P. aeruginosa* and *S. aureus*, but also the inhibition and eradication capability of limonene under shear stress. The protocol using this reactor has been approved by ASTM and these coupons where biofilm formation takes place under shear stress, are considered to provide the most conservative estimate of antimicrobial efficacy against biofilms, because they mimic the fluid flow conditions found in vivo. The biofilm formation on these coupons confirmed that not only are the types of material an essential prerequisite to decrease the spread of lethal infectious diseases in patients, but also microorganisms show less growth on steel both for mono- and polymicrobial biofilm.

The limonene added during the formation of biofilms at sub-MIC concentrations works very well in inhibiting both mono- and polymicrobial biofilms on all three materials, reducing their growth by about two logs. Of the same order of magnitude is the ability of limonene, added in continuous flow, to eradicate both mono- and polymicrobial mature biofilms on all three materials. In both cases, greater efficacy was observed in the polymicrobial biofilm on steel coupons. As for the evaluation, during the inhibition of biofilm formation, the expression of some genes related to the virulence of the two microorganisms was differently detected in mono- and polymicrobial biofilm. Therefore, in the mono-microbial biofilm of *P. aeruginosa,* the *pilA* and *flgK* genes were down-regulated (on polypropylene and steel) or not detected, and in the mono-microbial biofilm of *S. aureus,* the *eno* and *icaA* genes were upregulated (on polycarbonate and steel) or not detected. As demonstrated in other studies, transcriptional dynamics of locomotion related genes *flgK* and *pilA* of *P. aeruginosa* are involved in biofilm formation. Up-regulation of these genes enhanced biofilm formation and down-regulation inhibited biofilm; while in *S. aureus*, the *eno* and *icaA* genes are directly involved in switching from planktonic mode to biofilm mode of growth [20]. On the contrary, in polymicrobial biofilm we have had *pilA* and *flgK* genes up-regulated on all three materials while *eno* and *icaA* down-regulated in steel. This demonstrates and confirms that limonene is a natural compound that contrasts very well with the polymicrobial biofilm formed by *S. aureus* and *P. aeruginosa* and that steel is one of the surfaces on which this biofilm adheres less well.

These genes can be used as molecular markers for determining the resistance of bacteria against antibiotics.

## 5. Conclusions

These data showed that the limonene treatment expressed different levels of biofilm-forming genes, especially when both types of strains alone and together grew on different surfaces. Probably, for this reason, the limonene could be considered a method of infectious control showing an antipathogenic action in polymicrobial biofilm with an inhibitory effect on the production of virulence factors and, consequently, a decrease in biofilm formation. The four genes studied showed a different expression in the mono-microbial and polymicrobial biofilm, however demonstrating the ability of limonene to interfere with some virulence factors of the two microorganisms in a different way both from the point of view of the materials and the interaction between the two bacteria.

Our findings showed that limonene treatment is also very efficient when biofilm has been grown under shear stress causing significant and irreversible damage to biofilm structure.

Data indicated that limonene could be a future option to control polymicrobial biofilms grown on different materials assessing its ability to treat and/or prevent biofilm-related infections, and it could be an excellent candidate to be tested in vivo.

Therefore, the effectiveness of the sanitation procedures can be optimized by applying antimicrobial combinations with natural compounds (e.g., limonene), which would reduce the risk of the appearance of biocide resistant strains, could be more effective, and respectful of the environment and safe for health [37].

## Figures and Tables

**Table 1:** Gene names, acronym, primer name, sequence, and references of primers.

Gene Names	*Acronym*	Primer Name	Sequence (5′→3′)	References
*pilin gene*	*pilA*	*P. aeuriginosa_pilA_F*	GATCGAACTGATGATCGTGGT	Mirza et al., 2019 [20]
		*P. aeuriginosa_pilA_R*	GACATATGTTTCGGTCGCAGT	
*flagellar gene*	*flgK*	*P. aeuriginosa_flgK_F*	CGATACCGTCAACAAGCAACT	Mirza et al., 2019 [20]
		*P. aeuriginosa_flgK_R*	CTTGCTGGTATCGGTGATGTT	
*rpoS*	*rpoS*	*P. aeuriginosa_rpoS_F*	CTCCCCGGGCAACTCCAAAAG	Savli et al. 2003 [25]
		*P. aeuriginosa_rpoS_R*	CGATCATCCGCTTCCGACCAG	
*laminin-binding protein*	*eno*	*S. aureus_eno_F*	ACGTGCAGCAGCTGACT	Mirza et al., 2019 [20]
		*S. aureus_eno_R*	CAACAGCATCTTCAGTACCTTC	
*intercellular adhesion gene*	*icaA*	*S. aureus_icaA_F*	CCAGAAACATTGGGAGGTCTT	Mirza et al., 2019 [20]
		*S. aureus_icaA_R*	CCTTTTCGTTTTCATTGTGCT	
*16S Rrna*	*16S rRNA*	*S. aureus_16SrRNA_F*	TATGGAGGAACACCAGTGGCGAAG	Ogonowska and Nakonieczn, 2020 [26]
		*S. aureus_16SrRNA_R*	TCATCGTTTACGGCGTGGACTACC	

**Table 2:** Limonene MIC and MBC.

	MIC	MBC	MBC/MIC	
	mL/L		ratio	
*S. aureus*	20	>40	-	batteriostatic
*P. aeruginosa*	40	>40	-	batteriostatic

**Table 3:** Data of expression levels in *S. aureus* and *P. aeruginosa* biofilms individually and in combination, exposed to limonene at sub-MIC concentrations using polypropylene, polycarbonate, and steel coupons as substrate, were reported as a fold difference (in green down-expressed genes) from control (represented by biofilm untreated) after 48 h. Fold differences greater than ±1.5 were considered significant.

Gene	*Staphylococcus aureus + Pseudomonas aeruginosa*		
	Polypropylene	Polycarbonate	Steel
*pilA*	5.91	7.99	4.21
*flgK*	2.16	4.18	2.27
*eno*	0.50	−0.65	−6.98
*icaA*	0.49	−0.70	−7.50
	***Pseudomonas aeruginosa***		
	**Polypropylene**	**Polycarbonate**	**Steel**
*pilA*	−3.13	1.88	−3.13
*flgK*	−2.72	0.96	−2.72
	***Staphylococcus aureus***		
	**Polypropylene**	**Polycarbonate**	**Steel**
*eno*	−0.16	15.41	20.65
*icaA*	−2.38	10.58	22.84

## Data Availability

Not applicable.
